# The therapeutic role and potential mechanism of EGCG in obesity-related precocious puberty as determined by integrated metabolomics and network pharmacology

**DOI:** 10.3389/fendo.2023.1159657

**Published:** 2023-06-02

**Authors:** Qiuyun Gu, Lina Xia, Qiuju Du, Ying Shao, Jieyi He, Peiying Wu, Lingwei Liang, Xiuhua Shen

**Affiliations:** ^1^Department of Nutrition, Shanghai General Hospital, Shanghai Jiao Tong University School of Medicine, Shanghai, China; ^2^Department of Nutrition, School of Public Health, Shanghai Jiao Tong University School of Medicine, Shanghai, China; ^3^Department of Clinical Nutrition, College of Health Science and Technology, Shanghai Jiao Tong University School of Medicine, Shanghai, China

**Keywords:** EGCG, precocious puberty, obesity, metabolomics, network pharmacology

## Abstract

**Objective:**

(-)-Epigallocatechin-3-gallate (EGCG) has preventive effects on obesity-related precocious puberty, but its underlying mechanism remains unclear. The aim of this study was to integrate metabolomics and network pharmacology to reveal the mechanism of EGCG in the prevention of obesity-related precocious puberty.

**Materials and methods:**

A high-performance liquid chromatography-electrospray ionization ion-trap tandem mass spectrometry (LC-ESI-MS/MS) was used to analyze the impact of EGCG on serum metabolomics and associated metabolic pathways in a randomized controlled trial. Twelve weeks of EGCG capsules were given to obese girls in this trail. Additionally, the targets and pathways of EGCG in preventing obesity-related precocious puberty network pharmacology were predicted using network pharmacology. Finally, the mechanism of EGCG prevention of obesity-related precocious puberty was elucidated through integrated metabolomics and network pharmacology.

**Results:**

Serum metabolomics screened 234 endogenous differential metabolites, and network pharmacology identified a total of 153 common targets. These metabolites and targets mainly enrichment pathways involving endocrine-related pathways (estrogen signaling pathway, insulin resistance, and insulin secretion), and signal transduction (PI3K-Akt, MAPK, and Jak-STAT signaling pathways). The integrated metabolomics and network pharmacology indicated that AKT1, EGFR, ESR1, STAT3, IGF1, and MAPK1 may be key targets for EGCG in preventing obesity-related precocious puberty.

**Conclusion:**

EGCG may contribute to preventing obesity-related precocious puberty through targets such as AKT1, EGFR, ESR1, STAT3, IGF1, and MAPK1 and multiple signaling pathways, including the estrogen, PI3K-Akt, MAPK, and Jak-STAT pathways. This study provided a theoretical foundation for future research.

## 1 Introduction

Precocious puberty refers to the development of secondary sexual characteristics before the age of 8 years in girls and before the age of 9 years in boys (1). Precocious puberty can lead to accelerated skeletal maturation, advanced bone age, and early epiphyseal closure, all of which can impact final adult height. In addition, it may result in psychological issues or abnormal social behavior (2, 3). The long-term health consequences associated with early menarche include increased risk of obesity, type 2 diabetes, estrogen-dependent cancers, and cardiovascular events (3). It has been estimated that approximately one in five thousand children worldwide are affected by precocious puberty, with a markedly higher occurrence in girls than in boys, ranging from five to ten times more (2). Generally, girls are more likely to experience idiopathic precocious puberty, whereas approximately half of boys with precocious puberty have an identifiable cause. Thus, we focus on only girls in the present study (4). Currently, the primary recommended clinical treatment for precocious puberty is the utilization of gonadotropin-releasing hormone analog (GnRHa) (2). Although this treatment is considered safe and generally well -tolerated in children and adolescents (5), the GnRHa treatment cycle usually lasts longer than two years. Furthermore, the higher doses and considerable cost often impose significant psychological and financial burdens on the children’s family (6). Therefore, the prevention of precocious puberty has become an urgent public health goal.

Despite the etiology being incompletely understood, precocious puberty is thought to be associated with obesity, genes, lifestyle habits, environmental endocrine disruptors, and other factors (7, 8). Nutritional status and body fat mass are particularly vital factors contributing to precocious puberty (8, 9). Several research has indicated that obesity is closely linked to precocious puberty (8, 10). Therefore, preventing the occurrence of obesity has become a novel treatment strategy for preventing precocious puberty in girls. (-)-Epigallocatechin-3-gallate (EGCG) is the most abundant, bioactive, and extensively researched catechin in tea, with numerous studies verifying its efficacy in the prevention of obesity (11–13). Our research group found that EGCG has preventive effects on obesity-related precocious puberty (14), but the specific molecular mechanism remains to be elucidated.

Metabolomics is a comprehensive technique for monitoring the dynamics of endogenous small molecule metabolites and reflecting changes in metabolic pathways throughout the metabolic network in an organism (15). Network pharmacology is a widely utilized approach for determining the mechanism of drugs. High-throughput screening and analysis are employed to predict the complexity and integrality of the interactions between drugs and their targets and diseases and the related pharmacological mechanisms (16). In this study, the mechanism of action by which EGCG affected obesity-related precocious puberty was systematically investigated at the molecular level by integrated metabolomics and network pharmacology approach. The present research attempted to offer a theoretical foundation for further investigation into the treatment of obesity-related precocious puberty.

## 2 Materials and methods

### 2.1 Metabolomics analysis

Serum samples were derived from our prior randomized control trial (NCT03628937). Briefly, researchers assigned six to ten-year-old obese girls to two groups: placebo and EGCG. Twelve weeks of EGCG capsules (200mg, 50% EGCG) were given to girls in the EGCG group (n=18). The placebo group (n=16) received placebo capsules that appeared the same for twelve weeks. Detailed descriptions of the research design have been provided previously (14).

Serum metabolomics were analyzed using a high-performance liquid chromatography-electrospray ionization ion-trap tandem mass spectrometry (LC-ESI-MS/MS) in both positive and negative ion modes. Chromatographic conditions were based on the prior description (17).

Data analysis was carried out utilizing Majorbio Cloud (https://cloud.majorbio.com), based on the description given previously (17). In brief, the overall differences between the EGCG and Placebo groups were determined using orthogonal partial least squares discriminant analysis (OPLS-DA) after data preprocessing and annotation. Variable importance in the projection (VIP) values (>1.0) and statistical analysis (*P* < 0.05) were used to identify differential metabolites. The Kyoto Encyclopedia of Genes and Genomes (KEGG) and Human Metabolome Database (HMDB) were employed to identify and analyze implicated pathways associated with the metabolites.

### 2.2 Identification of EGCG targets

The PubChem database was employed to obtain simplified molecular-input line-entry system information and the 2D structure of EGCG (18), which were uploaded respectively to SwissTargetPrediction and PharmMapper Server for potential EGCG targets prediction (19). DrugBank was also used to identify EGCG targets (20). The UniProt database was utilized to correct all retrieved target names (21).

### 2.3 Identification of obesity and precocious puberty targets

Targets related to obesity and precocious puberty were found in three databases, including GeneCards (22), DisGeNET (23) and the Comparative Toxicogenomic Database (CTD) (24), using the key words “precocious puberty”, “early puberty” and “obesity”. To increase the credibility of analysis, this study included targets with a relevance score >1 in GeneCards, a gene-disease score >0.1 in DisGeNET, and an inference score >50 in CTD. We generated a Venn diagram through a VENNY 2.1 tool to identify common targets of EGCG, precocious puberty, and obesity.

### 2.4 The protein–protein interaction (PPI) network construction

PPI network was generated via uploading the intersecting targets of EGCG, precocious puberty, and obesity to the STRING database (25). Network visualization was conducted utilizing Cytoscape 3.7.2. software. To evaluate network topology, the CytoNCA plug-in was used, and the hub target genes were screened based on degree scores.

### 2.5 Gene Ontology (GO) and KEGG enrichment analyses

GO and KEGG pathway enrichment analyses were completed using the ClusterProfiler package of R 4.0.3 to elucidate the role of the common targets in gene function and signaling pathways (26). The GO analysis comprised of three primary components, including biological process (BP), cellular component (CC), and molecular function (MF). In order to guarantee the accuracy of the enrichment outcomes, the Benjamin-Hochberg method was employed for multiple testing correction. The adjusted P value < 0.05 was utilized as a threshold of significance for the enriched GO and KEGG terms for target genes. The GO enrichment analysis results were visualized through the online mapping platform Bioinformatics, and significant KEGG pathways (*P*<0.05) were visualized with OmicShare Tools.

### 2.6 Molecular docking

To validate the aforementioned results, molecular docking analysis was conducted to calculate the affinity of hub target gene products in the network. We selected hub target gene products and EGCG for molecular docking. From the PubChem database, three-dimensional (3D) structures of ligands were obtained (18). The RCSB database was utilized to acquire the 3D structures of receptors (27). Using mgltools_win32_1.5.6 software, ligands and receptors were repaired and saved as PDBQT files. The affinity of docking between EGCG and hub target proteins was determined utilizing AutoDock Vina 1.1.2 software. The molecular docking data were visualized utilizing PyMOL 2.3.

## 3 Results

### 3.1 EGCG alters the serum metabolome

**Figure 1:**
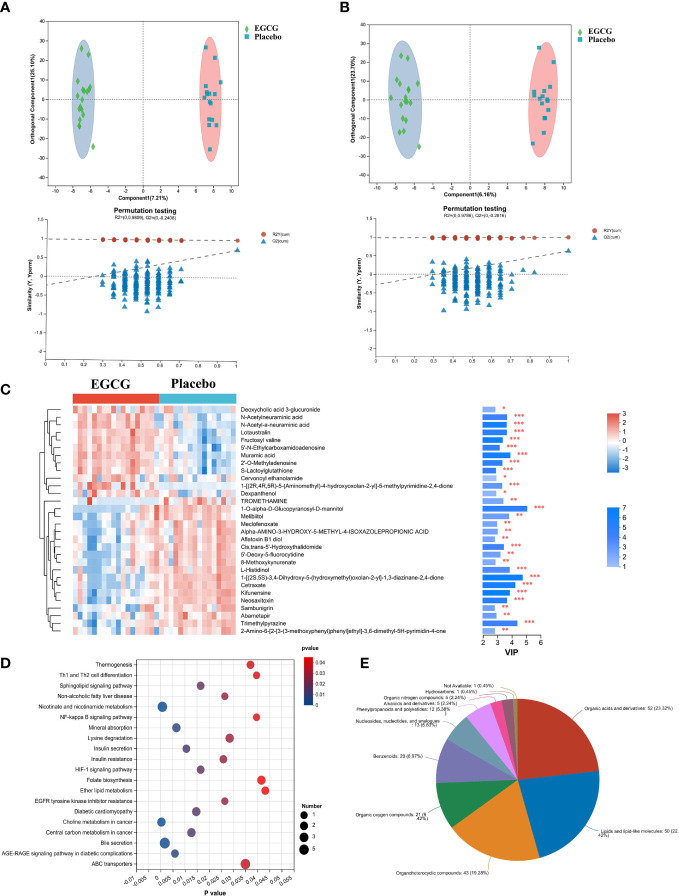
Multivariate statistical analysis of metabolite profiles in serum in obese girls OPLS-DA score plots of the EGCG group and Placebo group in positive mode **(A)** and negative mode **(B)**. **(C)** Heatmap of top 30 endogenous differential metabolites between two groups, *P < 0.05, **P < 0.01, ***P < 0.001. **(D)** KEGG signaling pathway enrichment analysis (*P* < 0.05). **(E)** Categories of endogenous differential metabolites.

This study applied metabolomic analysis to identify endogenous differential metabolites in serum of obese girls following intervention with EGCG. The OPLS-DA analysis revealed that serum samples from the EGCG and placebo groups were clearly separated in both positive and negative modes (**Figures 1A, B**), indicating significant differences between the two groups. The results of 200 permutations showed that there was no overfitting in the OPLS-DA model. In total, 234 endogenous differential metabolites were screened (**Supplementary Table 1**), and the top 30 metabolites were depicted in **Figure 1C**. **Figure 1D** illustrated endogenous differential metabolites enrichment pathways, mainly involving endocrine pathway (Insulin resistance, Insulin secretion, Non-alcoholic fatty liver disease and AGE-RAGE signaling pathway in diabetic complications), lipid metabolism (Sphingolipid metabolism and Ether lipid metabolism) and signal transduction (NF-kappa B and HIF-1 signaling pathway). With the help of the HMDB database, the differential metabolites between the EGCG and placebo groups were identified and classified according to the HMDB superclass. The majority of these endogenous differential metabolites were organic acids and derivatives (23.32%), and lipids and lipid-like molecules (22.42%) (**Figure 1E**). Based on these results, EGCG might have a vital regulatory effect on serum lipid metabolism, especially hormonal disorders, in obese girls.

### 3.2 Identification of potential targets of EGCG, obesity and precocious puberty

A total of 357 EGCG targets were obtained following the removal of duplicates from the Swiss Target Prediction, PharmMapper, and DrugBank databases (**Supplementary Table 2**). A total of 3447 targets of obesity were obtained after the merging of results from each database and the removal of duplicates (**Supplementary Table 3**). Similarly, 3994 targets of precocious puberty were collected by utilizing the key words “precocious puberty” and “early puberty” to search the databases (**Supplementary Table 4**).

### 3.3 PPI network of the potential therapeutic targets

**Figure 2:**
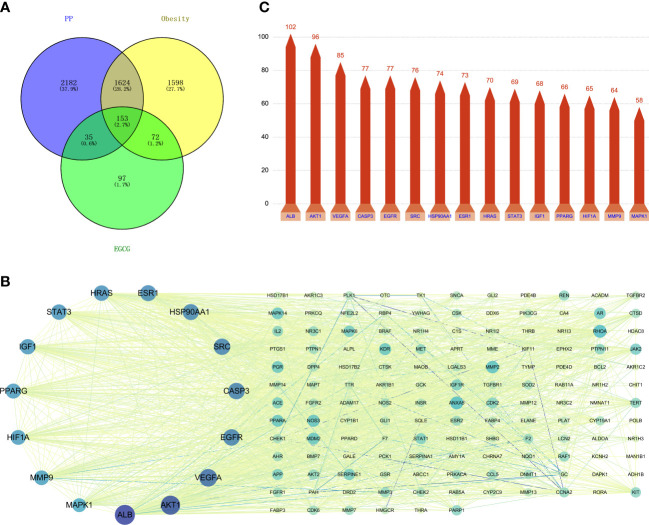
An analysis of potential therapeutic targets based on a Venn diagram and a PPI network **(A)** Venn diagram of the common targets of EGCG, obesity and precocious puberty. **(B)** Potential therapeutic targets in the PPI network. **(C)** The bar plot of the protein–protein interaction network.

At the intersection of the 357 EGCG targets, 3447 obesity targets and 3994 precocious puberty targets, we identified 153 common targets (**Figure 2A**, **Supplementary Table 4**). Subsequently, a PPI network was constructed for these 153 common targets (**Figure 2B**). A topological analysis was performed to identify hub target genes in this intricate biological network. We determined the top 15 hub target genes according to degree score: ALB, AKT1, VEGFA, CASP3, EGFR, SRC, HSP90AA1, ESR1, HRAS, STAT3, IGF1, PPARG, HIF1A, MMP9, and MAPK1 (**Figure 2C**). In light of these data, we speculated that EGCG might serve as a vital role in the treatment of obesity and precocious puberty through these 15 hub target genes and their products, which were utilized for the subsequent molecular docking analysis.

### 3.3 GO enrichment analysis

**Figure 3:**
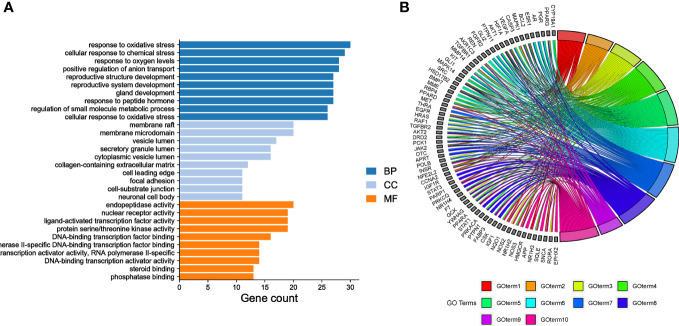
Gene Ontology (GO) enrichment analysis **(A)** GO enrichment analysis. The top 10 evidently enriched terms in each category. BP, biological process; CC, cellular component; MF, molecular function. **(B)** The top 10 enriched BP terms.

GO enrichment analysis was conducted to determine the relationships between the 153 common targets and diseases. A total of 2289 BP terms, 51 CC terms, and 163 MF terms were identified (*P*<0.05). The top ten significantly enriched terms from the three categories were output based on gene count (**Figure 3A**). The number of genes enriched in the BP category was the highest, indicating that EGCG mainly exerted its anti-obesity and anti-precocious puberty effects by regulating the BP of cells. The mechanisms indicated by the identified terms included the response to oxidative stress, cellular response to chemical stress, response to oxygen levels, positive regulation of anion transport, reproductive structure development, reproductive system development, gland development, response to peptide hormones, regulation of small molecule metabolic processes, and cellular response to oxidative stress. Thirteen of the fifteen hub genes were enriched in the top five enriched BPs, including AKT1, VEGFA, CASP3, EGFR, SRC, ESR1, HRAS, STAT3, IGF1, PPARG, HIF1A, and MAPK1 (**Figure 3B**). Based on the GO analysis, **Figure 3B** depicted the top 10 enriched BP terms.

### 3.4 KEGG enrichment analysis

**Figure 4:**
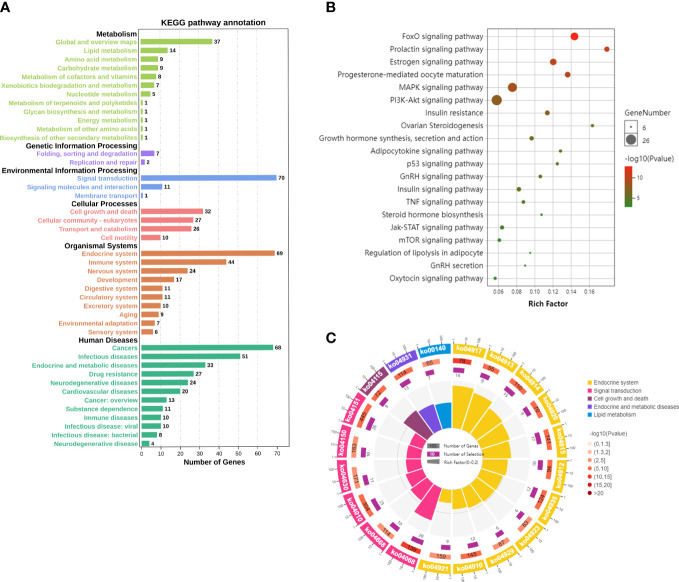
Kyoto Encyclopedia of Genes and Genomes (KEGG) pathway results **(A)** Statistical chart of the B-level classification of each pathway. **(B)** The top 20 significant pathways. Bubble size from large to small indicates the count of potential targets enriched in the pathway in descending order. Bubble color from red to green indicates the -log (p value) in descending order. **(C)** The top 20 significantly enriched differential pathway circle maps.

**Figure 5:**
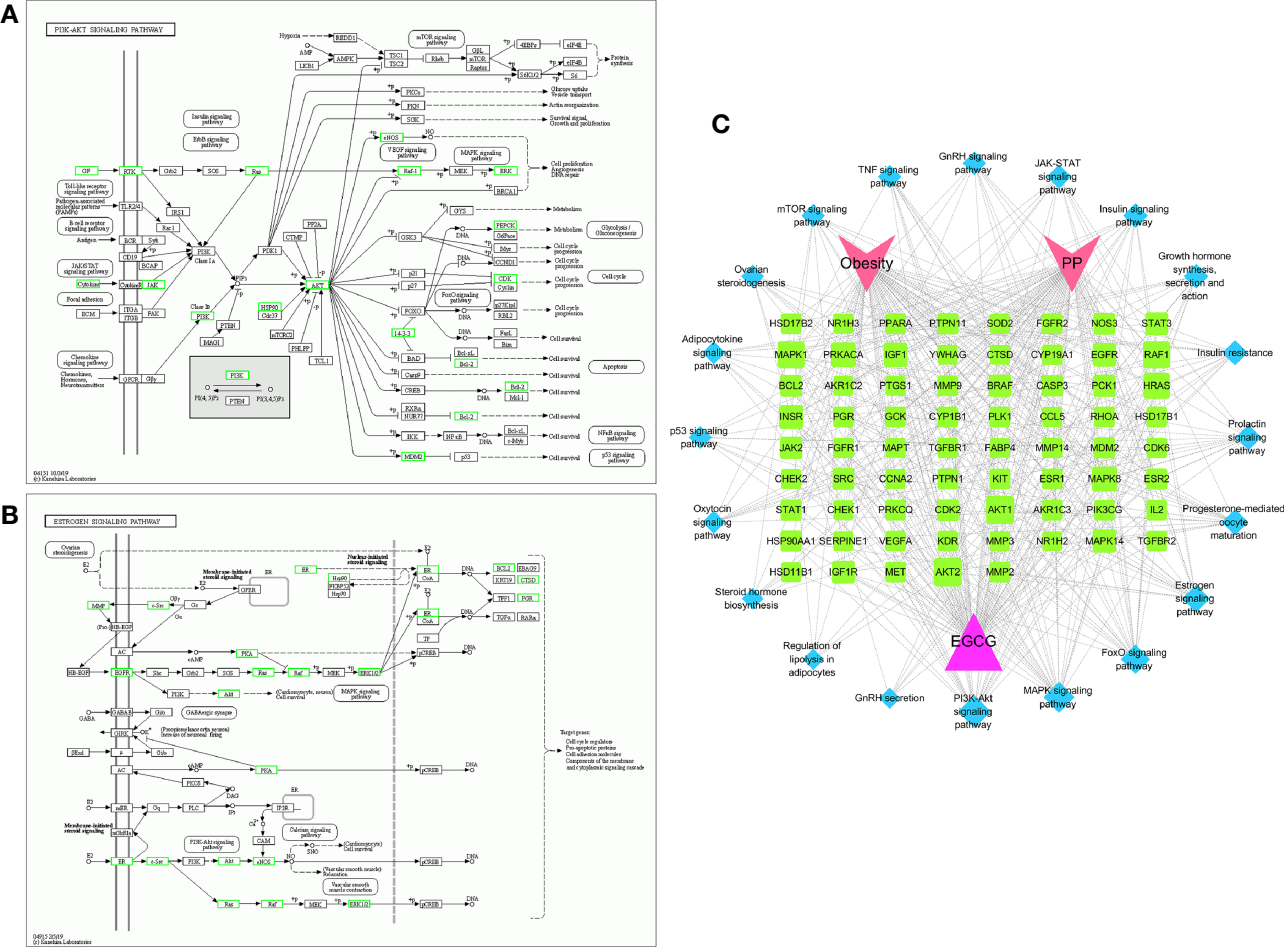
Potential therapeutic targets based on evidently enriched pathways The green nodes represent potential therapeutic targets of EGCG obesity and precocious puberty in PI3K-AKT pathway **(A)** and estrogen pathway **(B)**. **(C)** EGCG-target-pathway multiple interactive network.

The mechanism of EGCG in obesity and precocious puberty was further elucidated through KEGG pathway enrichment analysis. A total of 119 pathways with *P*<0.05 were obtained. The hub genes were predominantly enriched in metabolic pathways, environmental information processes (signal transduction), cellular processes (cell growth and death), and organismal systems (endocrine system) (**Figure 4A**). Twenty crucial pathways were identified following data screening (**Figures 4B, C**). The enriched genes were linked to endocrine-related pathways and processes, such as the estrogen signaling pathway, progesterone-mediated oocyte maturation, insulin resistance, and the prolactin signaling pathway. In addition, several of the identified signaling pathways were related to signal transduction, including the forkhead box O (FoxO) signaling pathway, mitogen-activated protein kinase (MAPK) signaling pathway, phosphatidylinositol 3-kinase (PI3K)-protein kinase B (Akt) signaling pathway, and Janus kinase (Jak)-signal transducer and activator of transcription (STAT) signaling pathway. A significant enrichment of the estrogen signaling pathway and PI3K-Akt signaling pathway was observed in the network (**Figures 5A, B**). Next, the component-target-pathway network diagram was created via Cytoscape 3.7.2 software, as shown in **Figure 5C**. This diagram demonstrated that 69 of the 153 possible targets were involved in the enrichment of the 20 significant pathways. Additionally, the pathways were interconnected, with certain targets overlapping and interacting with each other. These findings indicated that EGCG could influence multiple interacting pathways to interfere with obesity and precocious puberty.

### 3.5 Molecular docking results

**Figure 6:**
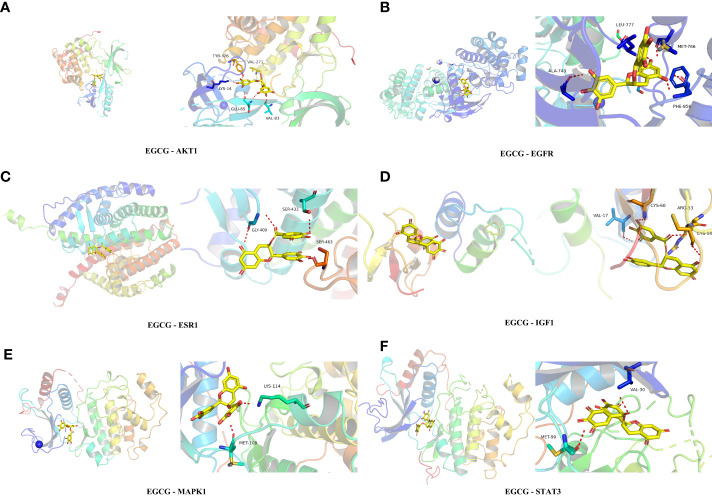
Molecular docking analysis **(A)** EGCG with AKT1. **(B)** EGCG with EGFR. **(C)** EGCG with ESR1. **(D)** EGCG with IGF1. **(E)** EGCG with MAPK1. **(F)** EGCG with STAT3.

**Table 1** presented the molecular docking outcomes of EGCG to the 15 hub target genes. The binding energies of all the simulations were <−7 kcal/mol, indicating strong binding affinity. **Figure 6** demonstrated the binding mode of EGCG with the hub targets, including AKT1, MAPK1, ESR1, EGFR, STAT3, and IGF1. These results suggested that EGCG had strong affinity for the 15 hub target gene products, which may be the primary targets of EGCG in preventing obesity-related precocious puberty.

**Table 1:** The molecular docking results of EGCG to the 15 hub target gene.

Target	PDB-ID	X	Y	Z	Affinity(kcal/mol)
AKT1	3O96	11.138	-13.389	15.819	-10.2
MAPK1	6G54	69.157	15.065	9.944	-9.2
EGFR	6DUK	39.051	89.525	-63.877	-9.1
PPARG	1PRG	36.77796	35.12079	39.30022	-9.1
ALB	1bke	30.74938	9.625792	29.45825	-8.8
CASP3	3H0E	24.89247	53.58088	12.29375	-8.5
HRAS	6MQT	-35.8738	-11.9815	50.90136	-8.4
HSP90AA1	1BYQ	40.44526	-46.8026	64.47693	-8.4
STAT3	5AX3	16.9628	-6.21055	-16.6396	-8.4
SRC	4U5J	-8.5193	58.86144	40.89687	-8.1
MMP9	1GKD	1.862463	32.99629	2.68274	-7.8
ESR1	1A52	107.008	15.86	99.456	-7.4
HIF1A	2ILM	19.7795	25.6017	28.3116	-7.3
IGF1	1H59	-2.63651	13.17597	21.02408	-7.3
VEGFA	4QAF	-21.7247	4.925429	2.271857	-7.1

## 4 Discussion

Precocious puberty is a common endocrine disease that is usually considered to be caused by the premature initiation of the hypothalamic-pituitary-ovarian axis (HPGA), resulting in the premature development of internal and external genitalia (28). Girls with precocious puberty are significantly more inclined to develop breast cancer, cardiovascular disease, and diabetes in adulthood (29). Our previous randomized controlled trial and animal experiments confirmed the significant ability of EGCG to prevent obesity-related precocious puberty (14, 30), but the underlying mechanism remained elusive. Therefore, this study applied integrated metabolomics and network pharmacology approach for the first time to elucidate the mechanism by which EGCG prevented obesity-related precocious puberty and to offer a theoretical foundation for forthcoming research. According to the results of integrated metabolomics and network pharmacology, 6 hub targets, including AKT1, EGFR, ESR1, STAT3, IGF1, and MAPK1, may play significant roles in the effects of EGCG on obesity-related precocious puberty.

The pathogenesis of precocious puberty is related to early initiation of the HPGA, and the pivotal factor in the HPGA initiation is the pulsatile secretion of gonadotropin-releasing hormone (GnRH) (31). Among the top 15 selected hub target genes was AKT1, one of three closely related serine/threonine protein kinases; AKT1 mainly regulates processes such as proliferation, metabolism, and cell survival (32). The phosphatase-binding function of AKT1 is required for IGF-1 and PI3K-mediated adipocyte differentiation, and IGF1 is crucial for preadipocyte proliferation, survival, and differentiation (33). IGF1 is a polypeptide comprised of 70 amino acids, whose function is analogous to that of insulin. IGF1 binds its receptor IGF-1R to activate the downstream PI3K/Akt signaling pathway. Mammalian target of rapamycin (mTOR) is a member of the PI3K-related kinase (PIKK family) and a downstream substrate of PI3K/Akt (34). Researchers have discovered that the hypothalamic IGF1/PI3K/Akt/mTOR signaling pathway regulates reproduction and development during puberty. By activating Kiss-1/GPR54, this pathway promotes GnRH release in the hypothalamus, thereby modulating adolescence (35).

Additionally, the estrogen receptor (ER) has been demonstrated to be strongly correlated with the pubertal development in children (36). ERα and ERβ are two isoforms encoded by ESR1 and ESR2, respectively, and are implicated in luteinization (37). A variety of cellular processes are regulated by SRC and HRAS, which are downstream of ESR1 in the estrogen pathway (38). MAPK1, also known as extracellular signal-regulated kinase 2 (ERK2), is a downstream effector of the epidermal growth factor receptor (EGFR) pathway. Activated ERK promotes follicle growth and ovulation via modulating the expression of gonadotropins LHβ and FSHβ (39). According to the KEGG pathway analysis, EGCG may affect estrogen signaling; ovarian steroidogenesis; insulin resistance; and other signaling pathways. These results supported the findings of our previous clinical study, which demonstrated that a 3-month intervention with 400 mg/d EGCG significantly reduced body fat percentage, ovarian volume and the number of follicles >4 mm in the ovaries of obese girls (14). This evidence suggested that EGCG might have considerable potential for preventing precocious puberty and improving obesity in girls. In addition, our prior experimental animal studies found that EGCG intervention remarkably decreased serum sex hormone levels in rats fed a high-fat diet (30). Moreover, evidence from *in vivo* studies suggests that the JAK/STAT pathway plays a crucial role in modulating GnRH neurons during puberty. Female GnRH neuron-specific JAK2 conditional knockout mice (JAK2 G-/-) shows reduced GnRH expression and neuronal activity. Furthermore, JAK2 G-/- female mice exhibits delayed puberty and reduced fertility (40). Therefore, EGCG may prevent obesity-related precocious puberty via the JAK2-STAT3 pathway. The present findings offered a novel foundation for the clinical application of EGCG and further related research.

Despite the findings of this study, the current study has certain limitations which should not be overlooked. Firstly, despite the advancements being made, there is still a lack of metabolomics data available for the precise identification of metabolites. Secondly, the sample size was too small. To improve this, it is essential to conduct future studies with a greater sample size of patients. Thirdly, due to the restrictions imposed by certain databases, it is not possible to retrieve all the active targets of EGCG, and the targets and pathways are interrelated and modulate each other. Further research is still required to gain a more comprehensive understanding of EGCG’s pharmacological effects and mechanisms in the prevention of obesity-related precocious puberty, which can be explored and confirmed through both *in vivo* and *in vitro* experiments. In the future, it will be necessary to thoroughly validate the details of omics data, including species markers that are distinct, predicted metabolic pathways, and potential functional interactions. Additionally, we can continue to explore the effects of EGCG on genes associated with estrogen, lipid metabolism, and energy metabolism, and further investigate the potential role of EGCG in regulating the endogenous metabolites of obesity-related precocious puberty. Furthermore, considering that the physiological association between EGCG and obesity-related precocious puberty is multifactorial, we will also use transcriptomic and proteomic technologies to reveal the correlation between EGCG and obesity-related precocious puberty at the gene and protein levels with the technical advantages of their high sensitivity and quantitative accuracy in the future research.

In summary, EGCG may serve a role in preventing obesity-related precocious puberty through targets such as AKT1, EGFR, ESR1, STAT3, IGF1, and MAPK1 that acted on multiple signaling pathways, including the estrogen, PI3K-Akt, MAPK, and Jak-STAT pathways. In this study, we investigated the mechanism by which EGCG prevented obesity-related precocious puberty through a network pharmacology approach and found that multiple targets and pathways were involved in the underlying mechanism. This study provided a theoretical foundation for subsequent research.

## Data availability statement

The datasets presented in this study can be found in online repositories. The names of the repository/repositories and accession number(s) can be found in the article/**Supplementary Material**.

## Ethics statement

The studies involving human participants were reviewed and approved by the Ethics Committee of Xinhua Hospital. Written informed consent to participate in this study was provided by the participants’ legal guardian/next of kin. The animal study was reviewed and approved by the Ethics Committee of Xinhua Hospital.

## Author contributions

XS and LL conceived and designed the study. QG, LX, QD, YS, and JH collected data. QG drafted the manuscript. PW provided major comments to the manuscript. All authors contributed to the article and approved the submitted version.

## Supplementary material

Supplementary Table 1 A total of 357 EGCG targets.

Supplementary Table 2 A total of 153 common targets in "Precocious puberty", "Obesity" and "EGCG".

Supplementary Table 3 Common targets in "Precocious puberty" and "Obesity".

Supplementary Data Sheet 1 Endogenous differential metabolites between two groups.
